# Symptomatic Mesenteric Panniculitis Associated With a Suspicious Cecal Lesion: A Case Report

**DOI:** 10.7759/cureus.109000

**Published:** 2026-05-16

**Authors:** El Mahdi Choukri, Zakaria Boulahcen, Othman El Belhadji, Zineb Moudafia, Siham Alaoui Rachidi

**Affiliations:** 1 Diagnostic and Interventional Radiology, Mohammed VI University Hospital, Tangier, MAR; 2 Radiology, Faculty of Medicine and Pharmacy, Abdelmalek Essaâdi University, Tangier, MAR

**Keywords:** acute abdominal pain, cecal wall thickening, computed tomography, mesenteric inflammation, mesenteric panniculitis

## Abstract

Mesenteric panniculitis is an uncommon inflammatory disorder involving the adipose tissue of the mesentery. Although it is frequently discovered incidentally on computed tomography (CT), it may occasionally present with nonspecific abdominal symptoms, making diagnosis challenging. We report the case of an 85-year-old woman who presented with a three-day history of acute abdominal pain. Contrast-enhanced abdominopelvic CT demonstrated increased attenuation of the mesenteric fat with small subcentimeter mesenteric lymph nodes, producing a “misty mesentery” appearance consistent with mesenteric panniculitis. The examination also revealed irregular circumferential thickening of the cecal wall, suspicious for an underlying malignant lesion. No bowel obstruction, hepatic metastasis, peritoneal nodularity, ascites, suspicious enlarged regional lymph nodes, or metastatic deposits in the visualized lung bases were identified on CT. The patient was referred for colonoscopic and histopathological evaluation; however, pathological confirmation was not documented in the available medical record. This case highlights the role of CT in identifying mesenteric panniculitis and detecting associated suspicious abdominal findings that may require targeted diagnostic workup. Awareness of this entity is important, particularly in symptomatic elderly patients, because careful CT assessment may identify additional clinically relevant abnormalities requiring further evaluation.

## Introduction

Mesenteric panniculitis is an uncommon, usually benign inflammatory condition involving the adipose tissue of the mesentery. It is considered part of the broader spectrum of sclerosing mesenteritis, a term that includes different pathological stages ranging from mesenteric lipodystrophy to retractile mesenteritis [1,2]. This terminology can be confusing because these entities may overlap clinically, radiologically, and histologically. The exact etiology remains uncertain, although several associations have been described, including prior abdominal surgery, trauma, autoimmune disorders, infection, and malignancy [1,3].

Reported CT prevalence is low, ranging from approximately 0.16% to 2.5% of abdominal CT examinations, with one classic CT study reporting a prevalence of 0.6% [1,4,5]. Mesenteric panniculitis is most often described in middle-aged and older adults, although sex distribution varies across series [1,4]. The clinical course is often benign or stable, although management depends on symptoms and associated findings [3,6].

Most cases are asymptomatic and are incidentally detected on imaging, particularly computed tomography (CT) [1,2]. When symptomatic, patients may present with nonspecific manifestations such as abdominal pain, abdominal discomfort, or digestive symptoms, which may mimic other abdominal disorders. CT plays a central role in diagnosis by demonstrating characteristic findings such as increased attenuation of mesenteric fat, small mesenteric lymph nodes, and the so-called “misty mesentery” appearance [1,7].

The relationship between mesenteric panniculitis and malignancy remains controversial. Some studies have reported an association with underlying or concomitant malignancies, particularly gastrointestinal and lymphoproliferative tumors, whereas others suggest that mesenteric panniculitis may simply represent an incidental imaging finding in patients undergoing CT for other reasons [2,5,8,9]. Therefore, mesenteric panniculitis should not be interpreted as evidence of malignancy by itself. However, when suspicious bowel wall thickening or other abnormal CT findings are present, targeted diagnostic evaluation is required.

We report a case of symptomatic mesenteric panniculitis identified on contrast-enhanced CT in an elderly patient, associated with irregular cecal wall thickening suspicious for an underlying malignant lesion. This case emphasizes the importance of careful abdominal CT assessment and cautious interpretation of the possible association between mesenteric panniculitis and malignancy.

## Case presentation

An 85-year-old woman presented to the emergency department with a three-day history of acute abdominal pain that persisted despite symptomatic treatment. The specific medications and doses used before admission were not documented in the available medical record. The pain was described as diffuse. No history of recent abdominal surgery or trauma was reported. Associated gastrointestinal symptoms, fever, weight loss, bowel habit changes, rectal bleeding, relevant comorbidities, and previous malignancy history were not documented in the available medical record.

On clinical examination, the patient was hemodynamically stable. Abdominal examination revealed diffuse tenderness without signs of peritonitis. Detailed laboratory values, including leukocyte count, C-reactive protein level, hemoglobin level, liver function tests, renal function tests, and tumor markers, were not documented in the available medical record.

**Figure 1:**
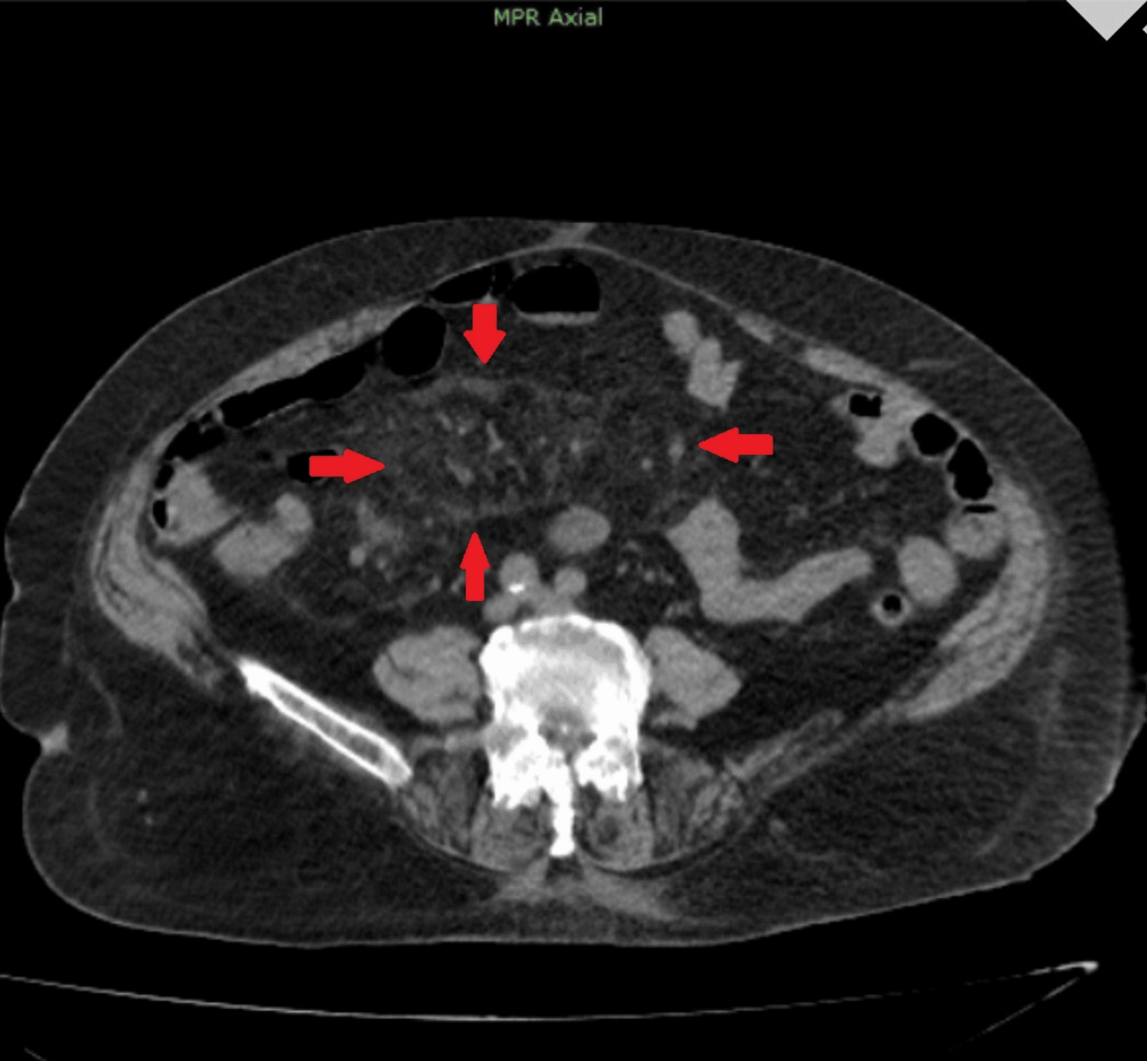
Axial contrast-enhanced CT image showing mesenteric panniculitis Axial contrast-enhanced CT image of the abdomen showing increased attenuation of the mesenteric fat, producing a “misty mesentery” appearance, with associated small subcentimeter mesenteric lymph nodes. These findings are consistent with mesenteric panniculitis. Red arrows indicate mesenteric inflammatory changes and small mesenteric lymph nodes. CT: computed tomography

A contrast-enhanced CT scan of the abdomen and pelvis was performed, which demonstrated increased attenuation of the mesenteric fat associated with small subcentimeter mesenteric lymph nodes, producing a “misty mesentery” appearance (Figure 1), consistent with mesenteric panniculitis. A definite fat ring sign or hyperattenuating pseudocapsule was not clearly identified on the available CT images.

**Figure 2:**
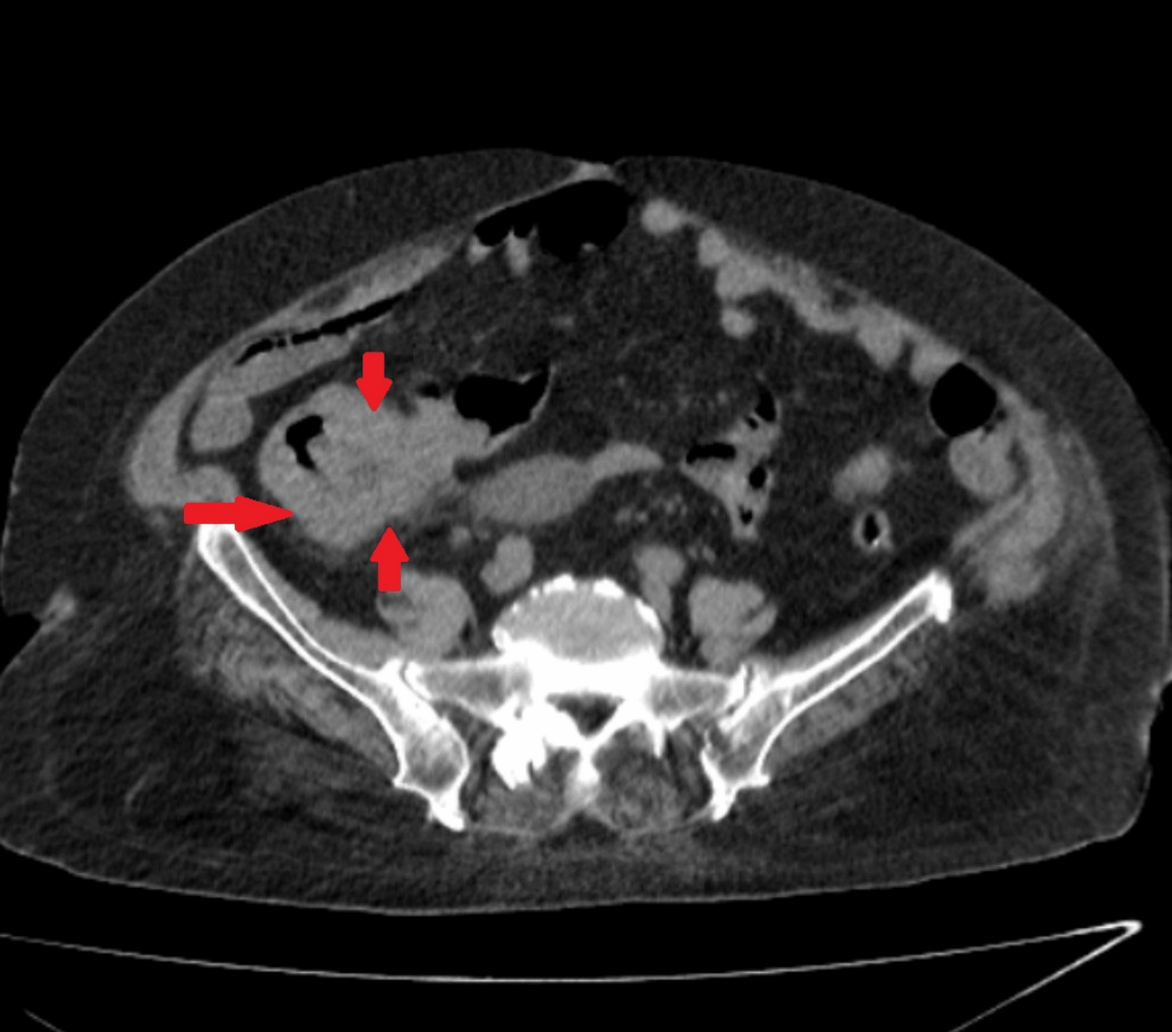
Axial contrast-enhanced CT image showing suspicious cecal wall thickening Axial contrast-enhanced CT image showing irregular circumferential thickening of the cecal wall, suspicious for an underlying malignant lesion. The red arrow indicates the abnormal cecal wall thickening. No bowel obstruction was identified on CT. CT: computed tomography

In addition, CT revealed irregular circumferential thickening of the cecal wall, suspicious for an underlying malignant lesion (Figure 2). The available imaging report did not provide precise measurements of the cecal wall thickening or lesion length. However, the lesion appeared irregular and circumferential, with adjacent fat infiltration and suspected luminal narrowing. No bowel obstruction, hepatic metastasis, peritoneal nodularity, or ascites, suspicious enlarged regional lymph nodes, or metastatic deposits in the visualized lung bases were identified on the available CT assessment.

**Figure 3:**
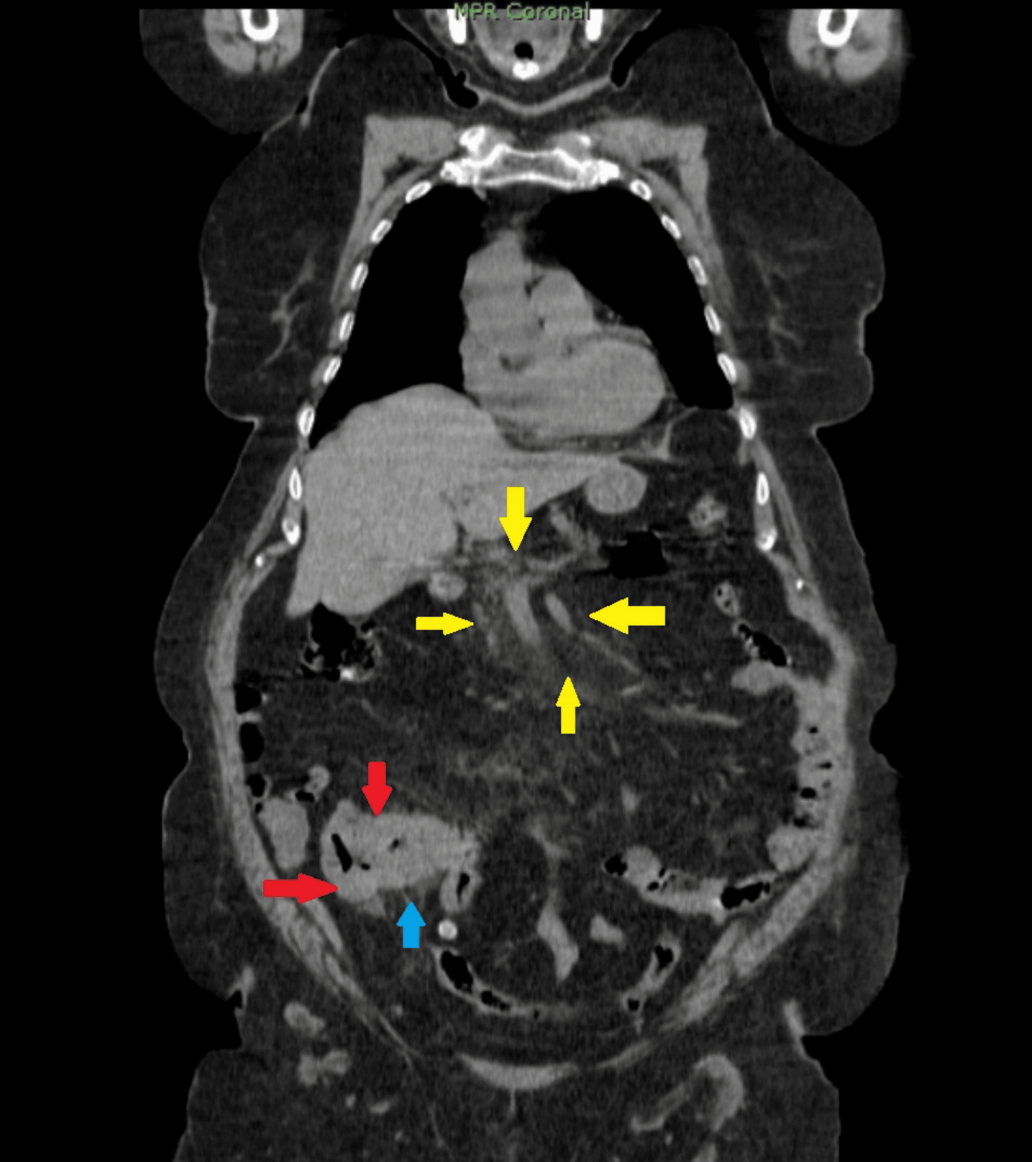
Coronal contrast-enhanced CT reconstruction showing the relationship between the cecal lesion and mesenteric panniculitis Coronal contrast-enhanced CT reconstruction showing irregular cecal wall thickening suspicious for an underlying malignant lesion, adjacent fat infiltration, and associated mesenteric panniculitis. Red arrow indicates the suspicious cecal wall thickening, blue arrow indicates adjacent fat infiltration likely related to the cecal lesion, and yellow arrow indicates mesenteric inflammatory fat changes consistent with mesenteric panniculitis. CT: computed tomography

Coronal reconstruction further illustrated the relationship between the suspicious cecal wall thickening, adjacent fat infiltration, and mesenteric panniculitis (Figure 3). The adjacent fat infiltration was considered more likely related to the suspicious cecal lesion, whereas the more central mesenteric fat stranding and small lymph nodes were compatible with mesenteric panniculitis.

Overall, the CT findings supported the diagnosis of mesenteric panniculitis associated with a suspicious cecal lesion. Because a malignant cecal process could not be excluded, colonoscopy with biopsy was recommended for definitive evaluation. The patient was referred for further gastroenterological assessment; however, colonoscopy findings, histopathological results, treatment course, and follow-up outcome were not documented in the available medical record.

## Discussion

Mesenteric panniculitis is most often detected incidentally on CT, but it may occasionally be encountered in symptomatic patients with nonspecific abdominal complaints. In such cases, radiologists should not only recognize the characteristic mesenteric findings but also carefully assess the entire abdomen for additional abnormalities that may explain the clinical presentation or require targeted investigation.

CT is the key imaging modality for diagnosis. Typical findings include increased attenuation of the mesenteric fat, small mesenteric lymph nodes, the “misty mesentery” appearance, preservation of fat around mesenteric vessels known as the fat ring sign, and, in some cases, a hyperattenuating pseudocapsule [7,10]. In the present case, the main CT findings were increased attenuation of the mesenteric fat with small subcentimeter mesenteric lymph nodes, consistent with mesenteric panniculitis. A definite fat ring sign or pseudocapsule was not clearly identified on the available images.

The main CT differential diagnoses of misty mesentery include lymphoma, peritoneal carcinomatosis, mesenteric edema, inflammatory or infectious mesenteric conditions, and hemorrhage [4,10]. Lymphoma may present with bulky lymphadenopathy or a confluent soft-tissue mass, whereas peritoneal carcinomatosis is usually associated with peritoneal nodules, ascites, or omental involvement. Mesenteric edema is generally related to systemic conditions such as heart failure, liver disease, hypoalbuminemia, or venous congestion. Recognition of these differential diagnoses is important because management differs substantially.

The association between mesenteric panniculitis and malignancy remains debated. Reported rates of associated malignancy vary widely across studies, partly because of differences in patient selection, CT indication, and whether previous, concomitant, or subsequent malignancies are included [1,5,8,9]. Several studies have reported an association with gastrointestinal and lymphoproliferative tumors, but this association does not prove causation [5,8,9]. Mesenteric panniculitis may represent a paraneoplastic inflammatory response in selected patients, but it may also be an incidental CT finding, especially in older patients undergoing imaging for unrelated reasons. Therefore, mesenteric panniculitis should not be interpreted as a direct marker of malignancy by itself.

In the present case, CT demonstrated both mesenteric panniculitis and irregular circumferential cecal wall thickening suspicious for an underlying malignant lesion. The suspicious cecal abnormality was not considered a proven malignancy because colonoscopic and histopathological confirmation was not documented in the available medical record. Therefore, the most appropriate interpretation is that CT identified mesenteric panniculitis associated with a suspicious cecal lesion requiring further evaluation, rather than a confirmed cecal neoplasm.

The adjacent fat infiltration was considered more likely related to the suspicious cecal lesion, while the more central mesenteric fat stranding and small lymph nodes corresponded to mesenteric panniculitis. This distinction is clinically relevant because bowel wall thickening with adjacent fat infiltration should prompt targeted evaluation of the bowel lesion, particularly in an elderly patient. In this setting, colonoscopy with biopsy represents the key diagnostic step to confirm or exclude malignancy.

Management of mesenteric panniculitis is not standardized and depends on symptoms, severity, and associated findings [6]. Asymptomatic cases often require no specific treatment. Symptomatic cases may be treated with anti-inflammatory or immunosuppressive therapy, including corticosteroids, tamoxifen, colchicine, or other agents in selected cases [3,6]. However, when CT demonstrates a suspicious bowel lesion, as in this case, the priority is diagnostic workup of the suspected neoplastic process rather than treatment of mesenteric panniculitis alone.

This case emphasizes the practical role of CT in symptomatic patients: recognizing mesenteric panniculitis, assessing for imaging features that support or challenge the diagnosis, evaluating relevant differential diagnoses, and identifying associated abnormalities that require further investigation. In elderly patients or when suspicious bowel wall thickening is present, careful correlation with symptoms, age-appropriate cancer screening, and targeted endoscopic evaluation are recommended.

## Conclusions

Mesenteric panniculitis is an uncommon condition that is often detected incidentally but may occasionally be encountered in symptomatic patients with nonspecific abdominal pain. CT plays a key role in diagnosis by demonstrating characteristic findings such as increased attenuation of mesenteric fat, small mesenteric lymph nodes, and the “misty mesentery” appearance. In this case, CT also identified irregular circumferential cecal wall thickening suspicious for an underlying malignant lesion. However, because colonoscopic and histopathological confirmation was not documented in the available medical record, the lesion should be considered suspicious rather than definitively malignant. This case highlights the importance of careful abdominal CT assessment, cautious interpretation of the debated association between mesenteric panniculitis and malignancy, and targeted endoscopic evaluation when suspicious bowel wall thickening is present.
